# Clinical and visual profile of ocular trauma in rural India: A cross-sectional descriptive study

**DOI:** 10.6026/973206300211804

**Published:** 2025-07-31

**Authors:** Abhay Kumar, Bharti Badlani, Anjali Singh, Pankaj Kushwaha

**Affiliations:** 1 Department of ENT, Chhindwara Institute of Medical Sciences, Chhindwara, Madhya Pradesh, India; 2 Department of Ophthalmology, Chhindwara Institute of Medical Sciences, Chhindwara, Madhya Pradesh, India

**Keywords:** Ocular trauma, rural health, visual acuity, open-globe injury, birmingham eye trauma terminology, India

## Abstract

Rural Indian communities are particularly vulnerable owing to agriculture-related hazards, delayed access to care, and limited eye-health literacy. Consecutive patients presenting with fresh or previously untreated mechanical ocular injury were enrolled. Clinical findings and initial/un-corrected visual acuity (VA) were recorded using the Birmingham Eye Trauma Terminology (BETT) and Ocular Trauma Score (OTS) frameworks. Community-level first-aid, rapid referral pathways and protective-eye-wear education substantially reduce blindness from eye injuries in rural settings.

## Background:

Ocular trauma accounts for an estimated 1.6 million cases of bilateral blindness and up to 19 million cases of unilateral visual loss worldwide [1]. In India, population-based surveys indicate a lifetime prevalence of 3-5 % for eye injuries, with higher rates in agrarian districts [1, 2- 3]. Multicentre analyses further demonstrate that young adult males sustain the greatest burden, often at the workplace or farm [2, 4]. The World Health Organization emphasises that 90 % of eye injuries are preventable through environmental modification and personal protective equipment [5, 6]. Despite these insights, regional heterogeneity persists regarding mechanisms of injury, clinical spectrum, and visual outcomes. Data specific to rural communities remain sparse, even though India's rural population constitutes nearly 65 % of the national demographic. Limited infrastructure, dependence on manual agricultural labour, and delays in accessing tertiary ophthalmic care may shape a distinct injury profile and outcome trajectory [5]. Standardised taxonomies such as BETT and quantitative prognostication tools like the Ocular Trauma Score (OTS) have enhanced the comparability of ocular-trauma research [7, 8, 9-10]. Yet few Indian studies utilise these frameworks comprehensively, and fewer still include both open- and closed-globe injuries across all age groups. Building such evidence is crucial for planning context-appropriate preventive and rehabilitative strategies. Therefore, it is of interest to report to delineate the demographic, clinical and visual profile of ocular trauma among patients attending a rural secondary-level eye hospital in northern India.

## Materials and Methods:

A hospital-based cross-sectional study was conducted at Chhindwara Institute of Medical Sciences, Chhindwara, MP, India, a tertiary care teaching institution, after approval from Institutional Ethics Committee [Ref. No. CIMS/EC/2024/14608]. All consecutive patients of any age and sex presenting with mechanical ocular trauma between 1 January 2023 and 31 December 2024 were screened. Exclusion criteria were: injury > 1 month old, chemical/thermal injury, prior ocular surgery, or refusal of consent. For minors, guardian consent was obtained. Approval was secured from the Institutional Ethics Committee [Ref. No. CIMS/EC/2024/14608]. The study adhered to the Declaration of Helsinki. Trained ophthalmology residents recorded demographics, injury setting, interval to presentation, and first-aid measures using a pre-tested proforma. Ocular examination included slit-lamp biomicroscopy, intra-ocular pressure (Tono-Pen®), dilated funduscopy, and B-scan ultrasonography when media opacity precluded retinal view. Injuries were coded per BETT categories. Visual acuity was measured with Snellen charts at 6 m (or child-appropriate Lea charts). The OTS was calculated from initial VA and presence of globe-rupture, endophthalmitis, perforation, retinal detachment or RAPD. Primary outcome was distribution of injury types and presenting VA. Secondary analyses explored associations between demographic/clinical variables and poor VA (≤20/200) using χ^2^ tests and multivariate logistic regression. Analyses were performed in Stata 17; p < 0.05 was significant.

## Results:

Four-hundred-twelve patients met inclusion criteria. Most injuries (64 %) occurred during field work involving hand tools or vegetative matter; household chores accounted for 18 %, road-traffic incidents 9 %, assaults 6 %, and recreational/sports 3 % (Figure 1 - see PDF). The modal age group was 21-30 years (3 %), followed by children ≤15 years (1 %). Males predominated (71 %; Table 1 - see PDF). Open-globe injuries (n = 90) were chiefly lacerations from metallic wires or thorny branches, whereas closed-globe injuries (n = 297) included contusions from sticks, stones and cattle hooves. Intra-ocular foreign bodies were detected in 12 %. The cornea (37 %) and sclera (19%) were the most common sites of impact (Table 2 - see PDF). Median delay to presentation was 19 h (IQR 6-48 h); 41 % sought care > 24 h post-injury. Presenting VA ranged from 20/20 to no light perception (NLP). Overall, 26 % presented with mild/no impairment (20/20-20/60), 36 % moderate (20/60-20/200), 34 % severe or worse (<20/200), and 4 % NLP (Figure 2 - see PDF). OTS categories 1 and 2 constituted 14 % and 22 % of eyes, respectively (Table 3 - see PDF). Multivariate analysis identified open-globe injury (adjusted OR 3.8, 95 % CI 2.1-6.7), presentation delay > 24 h (OR 2.4, 95 % CI 1.4-4.0) and age > 50 years (OR 1.9, 95 % CI 1.1-3.4) as independent predictors of poor VA. The model explained 41 % of variance (Nagelkerke R^2^ = 0.41) (Table 4 - see PDF).

## Discussion:

The present study provides contemporary data on ocular trauma within a rural Indian milieu, complementing prior work from teaching hospitals and urban eye-care networks [1, 2, 3-4]. Consistent with national and global trends, young working-age males predominated and agricultural activities were the single largest context for injury [2, 7]. The proportion of open-globe injuries (22 %) is comparable to reports from West Uttar Pradesh [1] and South Kerala [8], yet lower than figures from urban tertiary centres where high-velocity industrial wounds are common [9]. This underscores the need for context-specific preventive strategies.Our finding that presentation delay exceeding 24 h doubled the odds of severe visual loss echoes observations by Wisse *et al.* [10]. The median delay (19 h) reflects transport difficulties and low risk perception in rural districts. Community-based first-responder training and tele-ophthalmology triage could mitigate such delays. Visual outcome correlates strongly with structural damage; open-globe trauma was a fourfold predictor of poor VA. The OTS proved useful in quantifying injury severity, congruent with Kuhn's original validation 11[11] and subsequent Indian adaptations. However, nearly one-third of closed-globe injuries still presented with VA < 20/200. Early surgical management of lens opacities and secondary glaucoma screening should therefore integrate into rural trauma protocols. A notable 18 % of victims were children, often injured while assisting with farm chores without supervision. Eye-injury education should target both school curricula and farming cooperatives. The WHO's Integrated People-Centred Eye-Care agenda [10] advocates such inter-sectoral community engagement. The study's strengths include rigorous use of BETT/OTS, comprehensive enrolment over two calendar years, and multivariate modelling of risk factors. Limitations comprise its single-centre design, absence of follow-up visual outcomes, and potential referral bias towards more severe cases [12]. Future prospective cohorts should evaluate final VA at 6 months and cost-effectiveness of community prevention. Globally, the burden of eye injuries remains substantial; Clinical Medicine analysis estimated 6.3 million DALYs attributable to eye trauma annually [13]. Rural populations in low-resource settings shoulder a disproportionate share yet have the least access to emergency ophthalmic care. Our findings bolster the argument for strengthening district-level surgical capacity and subsidising protective eyewear for agricultural workers [14].

## Conclusion:

Ocular trauma in rural India is dominated by agriculture-related closed-globe injuries among young adult males, but open-globe wounds and delayed presentation drive the greatest visual loss. Systematic community education, timely referral pathways, and affordable protective eyewear could avert a significant proportion of rural blindness. Implementation of BETT/OTS protocols at secondary-level hospitals aids risk stratification and resource allocation. Thus, multicentre longitudinal studies evaluating intervention effectiveness are warranted to advance rural eye-injury control.
